# Patient’s perception of recovery after maxillary sinus floor augmentation with autogenous bone graft compared with composite grafts: a single-blinded randomized controlled trial

**DOI:** 10.1186/s40729-021-00379-y

**Published:** 2021-10-01

**Authors:** Thomas Starch-Jensen, Marianne Ahmad, Niels Henrik Bruun, Jonas Peter Becktor

**Affiliations:** 1 Department of Oral and Maxillofacial Surgery, Aalborg University Hospital, 18-22 Hobrovej, 9000 Aalborg, Denmark; 2 Department of Oral and Maxillofacial Surgery and Oral Medicine, Malmö University, Malmö, Sweden; 3 Unit of Clinical Biostatistics, Aalborg University Hospital, Aalborg, Denmark; 4 Department of Clinical Medicine, The Faculty of Medicine, Aalborg University, Aalborg, Denmark

**Keywords:** Alveolar ridge augmentation, Dental implants, Quality of life, Randomized controlled trial, Sinus floor augmentation

## Abstract

**Background:**

Autogenous bone graft is considered as the preferred grafting material for maxillary sinus floor augmentation (MSFA). However, harvesting of extraoral or intraoral autogenous bone graft is associated with risk of donor site morbidity and supplementary surgery. From a clinical and patient perspective, it would therefore be an advantage, if postoperative discomfort could be minimized by diminishing the need for autogenous bone graft harvesting. The objective of the present study was to test the hypothesis of no difference in patient’s perception of recovery after MSFA with autogenous bone graft from the zygomatic buttress (control) compared with 1:1 mixture of autogenous bone graft and deproteinized porcine bone mineral (DPBM) (Test I) or biphasic bone graft material (BBGM) (Test II). Sixty healthy patients were randomly allocated to either control or test groups. Oral Health-related Quality of Life (OHRQoL) was evaluated by Oral Health Impact Profile-14 (OHIP-14) at enrollment. Recovery was estimated by self-administrated questionnaires and visual analog scale assessing pain, social and working isolation, physical appearance, eating and speaking ability, diet variations, sleep impairment and discomfort after 1 week and 1 month. Descriptive statistics was expressed as mean with standard deviation (SD). Correlation between OHRQoL at enrollment and recovery were assessed by linear regression. *p*-value below 0.05 was considered significant.

**Results:**

Treatment satisfaction and willingness to undergo similar surgery were high in all groups. Average numbers of days with pain and sick leave were 3.5 (SD 3.9) and 0.5 (SD 1.2), respectively, with no significant difference between groups. Moreover, no significant difference in eating and speaking ability, physical appearance, work performance and sleep impairment were seen between groups. Mean OHIP-14 score at enrollment was 9.30 (SD 9.25) (control), 9.95 (SD 7.96) (Test I) and 8.15 (SD 9.37) (Test II), with no significant differences between groups. Impaired OHRQoL, gender or age seems not to predispose for delayed recovery or increased postoperative discomfort.

**Conclusions:**

MSFA with diminutive autogenous bone graft harvesting is associated with high patient satisfaction, limited postoperative discomfort and willingness to undergo similar surgery. Presurgical OHRQoL, gender or age seems not to be associated with impaired patient’s perception of recovery.

## Introduction

Vertical bone augmentation is often required prior to or in conjunction with implant placement in the posterior maxilla due to atrophy of the alveolar ridge and pneumatization of the maxillary sinus. Maxillary sinus floor augmentation (MSFA) applying the lateral window technique is the most frequently used method to increase the bone height of the posterior maxilla and high survival rates of suprastructures and implants as well as limited peri-implant marginal bone loss have been reported in several systematic reviews and meta-analyses regardless of used grafting material [1–6]. Autogenous bone graft from extraoral or intraoral donor sites are generally considered as the preferred grafting material for MSFA due to its osteoinductive, osteoconductive and osteogenic features [7, 8]. However, harvesting of autogenous bone graft is associated with supplementary surgery, risk of donor site morbidity and impaired postoperative Oral Health-related Quality of Life (OHRQoL) [9–12]. Bone substitutes alone or in combination with diminutive quantities of autogenous bone graft from intraoral donor sites are therefore used increasingly to decrease postoperative discomfort and simplifying the surgical procedure by diminishing the need for larger autogenous bone graft harvesting [2, 3, 13, 14]. However, the impact of diminutive autogenous bone graft harvesting from the surgical site on patient-reported outcome measures and OHRQoL in conjunction with MSFA are presently unknown.

Patient´s perception of recovery and assessment of OHRQoL in conjunction with MSFA are seldomly reported [15–21]. Previous studies have indicated that patient´s perception of recovery is influenced by patient-related predictors, incidence and severity of complications, presurgical expectations, past dental experiences as well as psychological and psychosocial factors [22–26]. Presurgical psychologic distress, high level of anxiety and impaired perception of OHRQoL seem to have a negative impact on patient´s perception of recovery [22–26]. However, association between impaired presurgical OHRQoL and patient’s perception of recovery following MSFA have never been assessed. Therefore, the objective of the present single-blinded randomized controlled trial was to test the hypothesis of no difference in patient’s perception of recovery after MSFA with autogenous bone graft compared with 1:1 mixture of autogenous bone graft and deproteinized porcine bone mineral (DPBM) or biphasic bone graft material (BBGM) using validated self-administrated questionnaires including a correlation with presurgical assessment of OHRQoL.

## Material and methods

**Fig. 1:**
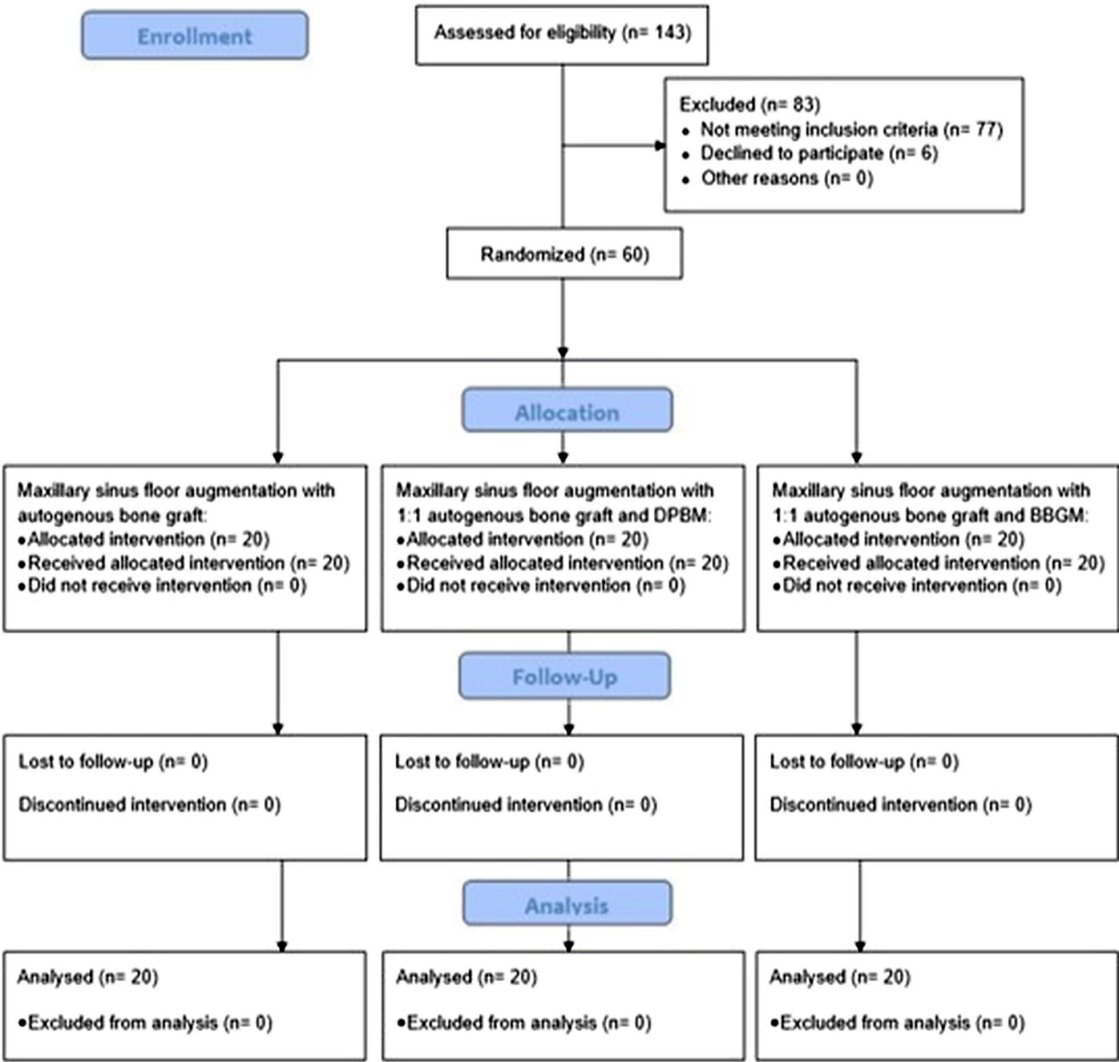
CONSORT flow diagram

The present study was designed as a parallel single-blinded randomized controlled trial. The study protocol was prepared in accordance with guidelines for reporting randomized controlled studies (CONSORT) (http://www.consort-statement.org/) and the study flowchart is outlined in Fig. 1. The protocol was registered in Clinicaltrials.gov (registration no: NCT04749953) and approved by The North Denmark Region Committee on Health Research Ethics (approval no: N-20170087) and The Swedish Ethical Review Authority in Lund, Sweden (approval no: Dnr. 2018/297).

Based on sample size calculation and assuming a 15% drop-out rate, it was planned to include 20 patients for each treatment group, in order to detect a 20% difference with a standard deviation of 15 between the three groups in long-term peri-implant marginal bone loss, with a power of 0.8 and a significance level equal to 0.05.

Patients were recruited by public invitation through Facebook or admitted to the Department of Oral and Maxillofacial surgery, Aalborg University Hospital, Aalborg, Denmark or Department of Oral and Maxillofacial Surgery and Oral Medicine, Malmö University, Malmö, Sweden for implant placement in the posterior maxilla. Candidates were screened for inclusion and exclusion criteria at enrollment (Table 1). The residual bone height in the posterior maxilla was estimated by cone beam computed tomography. Included patients received written as well as verbal information regarding the study protocol and signed an informed consent form before initiating the study. A total of 60 partially edentulous healthy patients with a missing posterior maxillary tooth were included and randomly allocated into three groups of 20 patients. In each group, 10 patients were treated in Aalborg and Malmö, respectively. A computer-aided block randomization was used to allocate included patients into three groups of same size. The randomized code was available in closed identical non-transparent sealed envelopes. Immediately before surgery, patients were randomly assigned to either autogenous bone graft [Control Group, (CG)], 1:1 mixture of autogenous bone graft and DPBM [Test Group I, (TI)] or 1:1 mixture of autogenous bone graft and BBGM [Test Group II, (TII)]. Patients were blinded to which treatment group they were assigned.

**Table 1:** Inclusion and exclusion criteria

Inclusion criteria
> 20 years
Missing posterior maxillary tooth/teeth for more than 4 months
Residual alveolar bone height at implant site (as measured on a cone beam computed tomography) ≥ 3 mm and ≤ 7 mm
Width of the alveolar ridge ≥ 6.5 mm
Mandibular occluding teeth
Able to understand and sign informed consent
Exclusion criteria
Contraindications to implant therapy
Full mouth plaque score > 25%
Progressive marginal periodontitis
Acute infection in the area intended for implant placement
Parafunction, bruxism, or clenching
Psychiatric problems or unrealistic expectations
Heavy tobacco use define as > 10 cigarettes per day
Current pregnancy at the time of recruitment
Physical handicaps that would interfere with the ability to perform adequate oral hygiene
Inability or unwillingness to regularly attend the scheduled follow-up visits

### Surgical procedure

One hour prior to surgery, patients were pre-medicated with analgesics involving 400 mg Ibuprofen (Burana, Teva, Denmark; Ipren, McNeil, Sweden) and 1000 mg paracetamol (Pamol, Takeda Pharma A/S, Denmark; Panodil, Perrigo, Sweden) and prophylactic antibiotic therapy including 2 g amoxicillin (Imadrax, Sandoz, Denmark; Amimox, Meda, Sweden) or clindamycin 600 mg (Dalacin, Alternova, Denmark; Dalacin, Pfizer, Sweden) if allergic to penicillin. All patients rinsed with 0.12% chlorhexidine solution for one minute immediately before surgery. The surgical procedures were conducted by two experienced and calibrated surgeons (TSJ, Aalborg and JPB, Malmö) in local anesthesia using Lidocaine (2%) with 1:200,000 adrenaline (Xylocaine, Amgros I/S, Denmark; Xylocaine Dental Adrenalin, Dentsply De Trey, Gmbh, Germany). A horizontal crestal incision was made from tuber maxillae with an anteriorly vertical releasing incision. A full thickness mucoperiosteal flap was raised to expose the lateral maxillary sinus wall. Autogenous bone graft was harvested with a disposable, cortical bone collector (Curved SafeScraper^®^, Fischer Medical ApS, Glostrup, Denmark; SafeScraper^®^, twist-curved, Meta, Reggio Emilia, Italy) from the zygomatic buttress area. Customized stainless-steel cups (1.0 cm^3^) were used to estimate equivalent amount of autogenous bone graft. The different compositions of the grafting material consisted of: (1) 2.0 cm^3^ autogenous bone graft (CG), (2) mixture of 1.0 cm^3^ autogenous bone graft and 1 ml DPBM (Symbios Xenograft Granules, 1.0–2.0 mm, Dentsply Sirona, Implants, Mölndal, Sweden) (TI), and (3) mixture of 1.0 cm^3^ autogenous bone graft and 1 ml BBGM (Symbios Biphasic Bone Graft Material, 1.0–2.0 mm, Dentsply Sirona Implants, Mölndal, Sweden) (TII). The different compositions of the grafting material were soaked in autogenous blood from the surgical site until use. A 1 × 1 cm window to the maxillary sinus was created with metal and diamond burrs. The Schneiderian membrane was carefully elevated from the sinus floor as well as the lateral sinus wall and displaced dorsocranially with blunt dissector. If a minor perforation of the Schneiderian membrane occurred, it was securely covered by a resorbable pre-hydrated collagen membrane (Symbios Collagen Membrane pre-hydrated, Dentsply Sirona Implants, Mölndal, Sweden). If the Schneiderian membrane was largely perforated with a major communication to the sinus cavity, the patient was withdrawn from the study. An implant bed was successively prepared on the top of the alveolar crest following manufactory’s recommendations. A straight 13-mm implant (OsseoSpeed EV, Astra Tech Implant System, diameter 3.6, 4.2, or 4.8, Dentsply Sirona Implants, Mölndal, Sweden) was inserted including a cover screw. The grafting material was gently packed around the exposed implant surface protruding into the maxillary sinus cavity, from the floor to underneath the Schneiderian membrane. The created window to the maxillary sinus was covered with a passively adapted Symbios pre-hydrated collagen membrane (20 mm × 30 mm, Dentsply Sirona Implants, Mölndal, Sweden). Periosteum and mucosa were sutured with Vicryl 4-0 (Ethicon FS-2, Ethicon, St-Stevens-Woluwe, Belgium). No provisional restoration was inserted during the healing period. Patients were instructed to rinse with 0.12% chlorhexidine solution twice a day until suture removal had taken place after 7–10 days. Moreover, patients were instructed to avoid any physical activity that could abruptly raise or lower pressure in the sinus cavity as well as avoiding vigorous mouth rinsing, smoking and touching the gums for at least 10 days following surgery. Postoperative analgesic was prescribed involving 400 mg Ibuprofen, 1 tablet 3 times daily and 500 mg paracetamol, 2 tablets 4 times per day, as long as required. All patients were prescribed postoperative antibiotics involving 800 mg phenoxymethylpenicillin (Primcillin, Meda, Denmark; Kåvepenin, Meda, Sweden), 2 tablets 3 times daily for 7 days. In case of penicillin allergy, 300 mg Clindamycin, 1 tablet 3 times daily for 7 days was used.

### Patient’s perception of recovery and patient-reported outcome measures

Oral Health Impact Profile-14 (OHIP-14) was used to assess OHRQoL at enrollment. OHIP-14 is organized into seven conceptual dimensions including functional limitation, physical discomfort, psychological discomfort, physical disability, psychological disability, social disability and handicap [27, 28]. Two items were used to measure each dimension and consequently the questionnaire consists of 14 items (Table 2). Response format was as follows: Very often = 4; Fairly often or many times = 3; Occasionally = 2; Hardly ever or nearly never = 1; Never/I don’t know = 0. The OHIP-14 scale ranged from 0 to 56 and dimension score ranged from 0 to 8. The values of the 14 items and each dimension were summed to calculate the OHIP-14 severity score, with higher scores indicating poorer OHRQoL.

**Table 2:** OHIP-14 questionnaire

OHIP-14- dimension score	Question	
Functional limitation	Q1 Q2	Have you had trouble pronouncing any words because of problems with your teeth, mouth or dentures?
		Have you felt that your sense of taste has worsened because of problems with your teeth, mouth or dentures?
Physical pain	Q3 Q4	Have you had painful aching in your mouth?
		Have you found it uncomfortable to eat any foods because of problems with your teeth, mouth or dentures?
Psychological discomfort	Q5 Q6	Have you been self-conscious because of your teeth, mouth or dentures?
		Have you felt tense because of problems with your teeth, mouth or dentures?
Physical disability	Q7 Q8	Has your diet been unsatisfactory because of problems with your teeth, mouth or dentures?
		Have you had to interrupt meals because of problems with your teeth, mouth or dentures?
Psychological disability	Q9 Q10	Have you found it difficult to relax because of problems with your teeth, mouth or dentures?
		Have you been a bit embarrassed because of problems with your teeth, mouth or dentures?
Social disability	Q11 Q12	Have you been a bit irritable with other people because of problems with your teeth, mouth or dentures?
		Have you had difficulty doing your usual jobs because of problems with your teeth, mouth or dentures?
Handicap	Q13 Q14	Have you felt that life in general was less satisfying because of problems with your teeth, mouth or dentures?
		Have you been totally unable to function because of problems with your teeth, mouth or dentures?

Patient’s perception of recovery including pain, oral function impairments, general activity and other symptoms was assessed after 1 week. A self-administrated questionnaire examined social isolation, working isolation, physical appearance and mean duration of the quality of life alterations as well as questions whether they would undergo similar treatment again, if needed or if they would recommend this treatment to a friend or a relative, if indicated. Response format was yes or no. Eating ability and diet variations, speaking ability noticed, sleep impairment, pain and discomfort at suture removal was also examined through self-administrated questionnaire after 1 week. Each item was evaluated by means of a four-point Likert-type rating scale. Response format was as follows: Not at all = 0; close to normal = 1; almost normal = 2; a little = 3. The rating score was calculated, with higher score indicating poorer patient recovery. Self-administrated questionnaire also examined how many days they had been on sick leave or been off work, had eating and speech difficulties, and how long their sleep and physical activity had been affected.

Patient’s perception of recovery was also examined by a self-administrated questionnaire after 1 month and supplemented by a 100 mm (0 = minimal to 100 = maximum) visual analogue scale (VAS) assessing pain, social isolation, working isolation, eating ability, speaking ability and sleep impairment.

Instructions for completing OHIP-14, self-administrated questionnaires and VAS were explained in detail to all patients. Patients completed the questionnaires by themselves, to prevent being influenced by the surgeons or nurses’ opinions and wills. Moreover, in order not to influence the compilation of the questionnaire, patients were not informed about their allocation group.

Intra- and postoperative complications including perforation of the Schneiderian membrane, bleeding, infection, wound dehiscence, nasal bleeding, or other adverse events regarding implant or grafting material were also registered.

### Correlation of patient’s perception of recovery and Oral Health-related Quality of Life

Impaired OHRQoL at enrollment was correlated to the self-administrated questionnaires assessing patient’s perception of recovery after 1 week and 1 month. OHIP-14 item score of 10 or more was considered as impaired preoperative OHRQoL. Moreover, OHIP-14 item score was correlated to age, gender, number of days with pain or on sick leave.

### Statistical analyses

Data management and analysis was conducted using STATA (Data analysis and statistical software, version 16, StataCorp P, Texas, USA). Mean and standard deviations were reported when variables were considered continuous, e.g., scores and Likert scales. Continuous variables were compared by Anova and *t*-test, while gender was analyzed by Fisher’s exact test. Categorical variables were reported by counts and percentages. Level of significance was 0.05. Dependency between OHIP-14 and age, gender, number of days with pain or on sick leave were analyzed by linear regression.

## Results

Patient demographics and frequency of complications in each group are outlined in Table 3. There were no significant differences between the groups according to gender (*p* = 0.72), age (*p* = 0.81), residual alveolar bone height (*p* = 0.77), or width of the alveolar process (*p* = 0.94).

**Table 3:** Demographic characteristics of the included patients

	Autogenous bone graft	1:1 autogenous bone graft and DPBM	1:1 autogenous bone graft and BBGM	*p*-value
Number of patients	20	20	20	
Gender (male/female)	9/11	10/10	7/13	0.72
Age at the time of surgery, mean (SD)	55.5 year (SD 13.1)	57.3 year (SD 14.4)	58.4 year (SD 15.5)	0.81
Smoking habits	1	2	1	
Residual alveolar bone height (mm) at implant site, mean (SD)	4.7 (1.1)	4.9 (1.4)	4.6 (1.5)	0.77
Width of the alveolar ridge (mm) at implant site, mean (SD)	10.0 (2.2)	9.9 (1.8)	9.8 (1.5)	0.94
Location of missing tooth (first premolar)	1	2	1	
Location of missing tooth (second premolar)	3	6	2	
Location of missing tooth (first molar)	16	12	16	
Location of missing tooth (second molar)	0	0	1	
Patient drop-out before surgical intervention	0	0	0	
Number of implants with 3.6 mm diameter	6	2	1	
Number of implants with 4.2 mm diameter	9	6	12	
Number of implants with 4.8 mm diameter	5	12	7	
Intraoperative perforation of the Schneiderian membrane	4	4	1	
Minor epistaxis during the first postoperative days	4	0	1	
Hematoma	1	1	1	
Postoperative infection	0	0	1	

*BBGM* biphasic bone graft material, *DPBM* deproteinized porcine bone mineral, *SD* standard deviation

A minor perforation of the Schneiderian membrane occurred in nine patients (15%), which were successfully covered by a resorbable pre-hydrated collagen membrane. Healing was uneventful in 51 patients (85%) and no implant losses were observed. One patient (TII) presented with infection after 1 week, which was effectively treated with antibiotic for additional 7 days. Five patients described minor epistaxis during the first postoperative days and three patients presented with a large hematoma after 1 week. All included patients attended postsurgical examinations and completed self-administrated questionnaires and VAS.

**Fig. 2:**
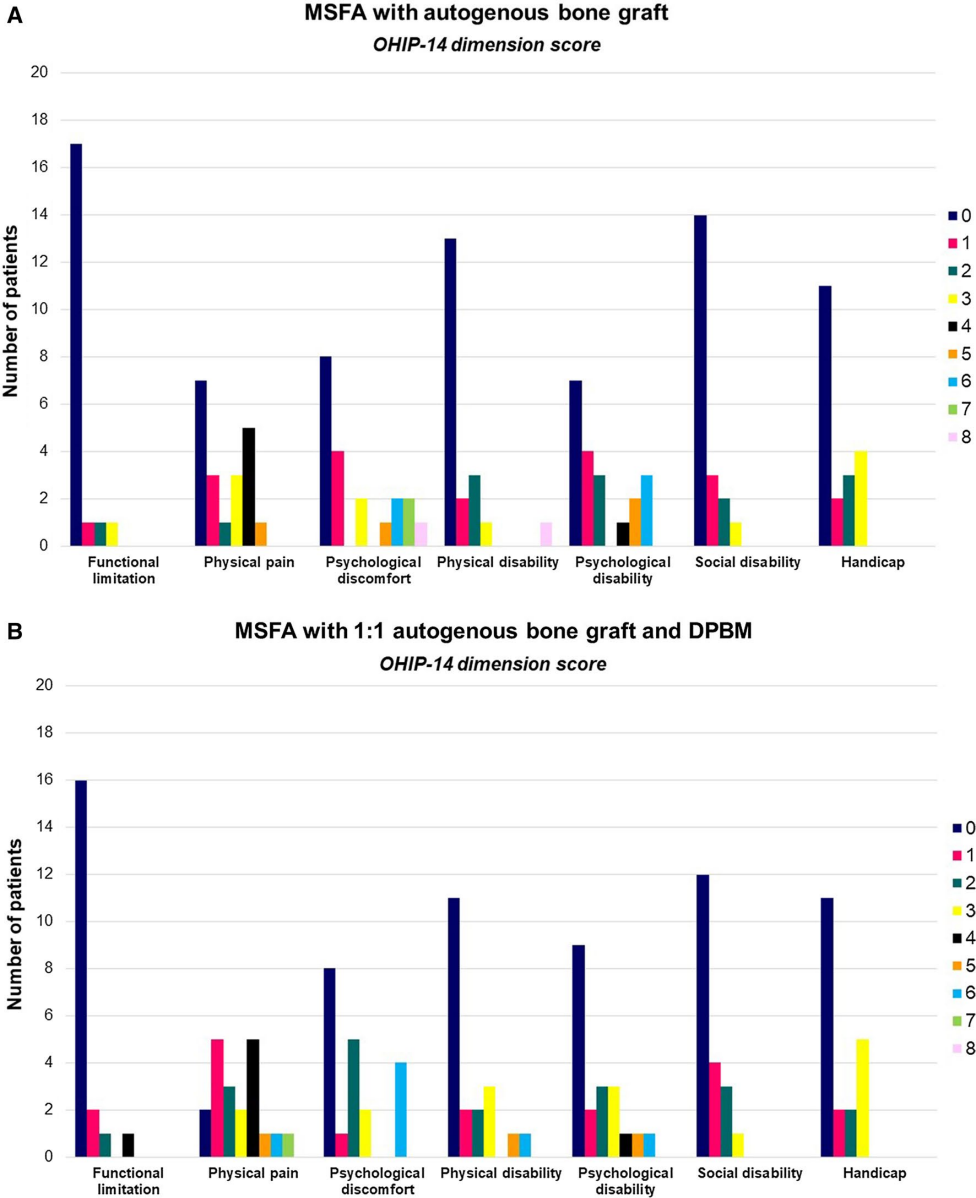

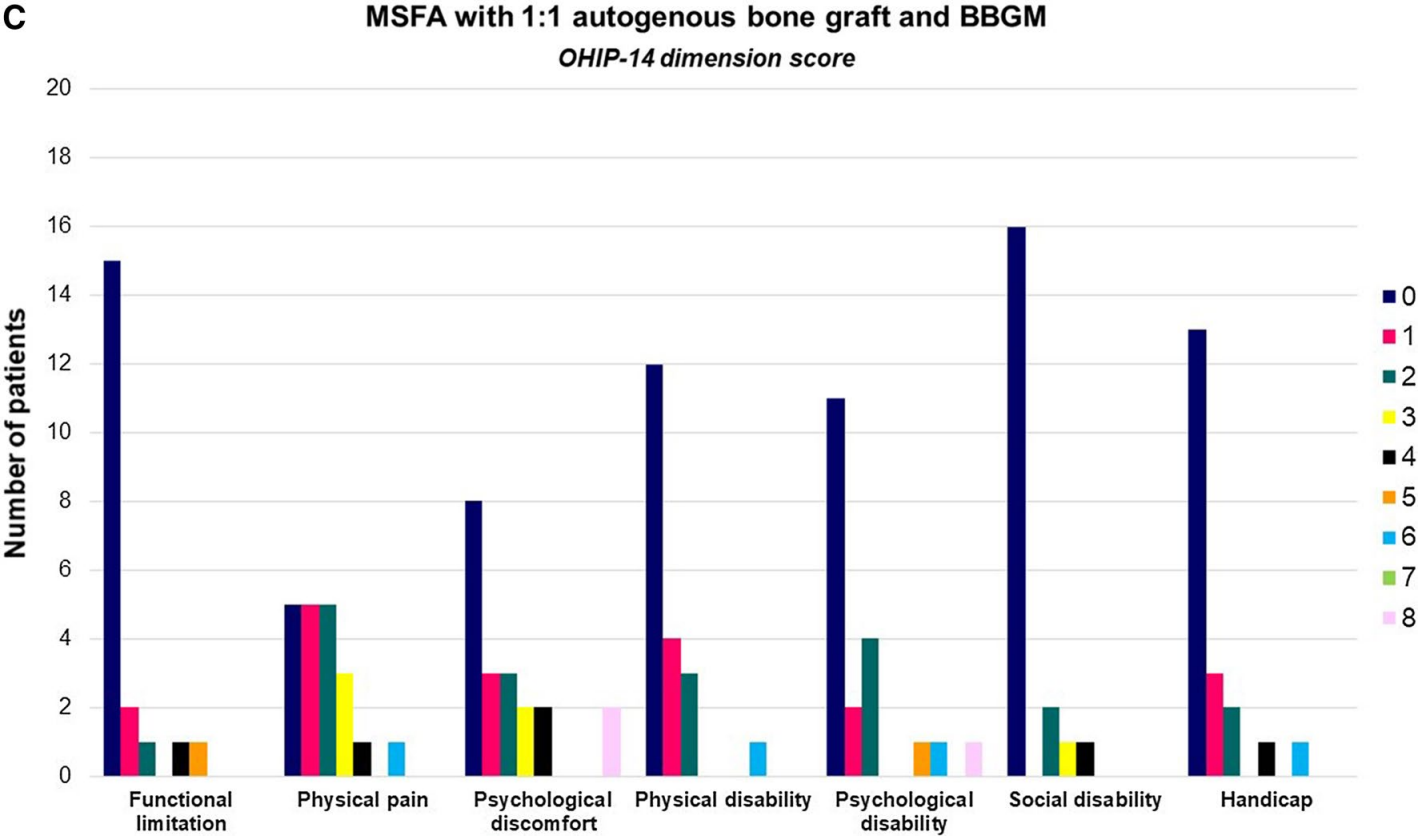
**A** OHIP-14 subscale dimension score after maxillary sinus floor augmentation with autogenous bone graft, at enrollment. **B** OHIP-14 subscale dimension score after maxillary sinus floor augmentation with 1:1 mixture of autogenous bone graft and deproteinized porcine bone mineral (DPBM), at enrollment. **C** OHIP-14 subscale dimension score after maxillary sinus floor augmentation with 1:1 mixture of autogenous bone graft and biphasic bone graft material (BBGM), at enrollment

OHIP-14 score at enrollment was 186 (CG), 199 (TI), and 163 (TII) (Table 4). Mean OHIP-14 score for each patient was 9.30 ± 9.25 (CG), 9.95 ± 7.96 (TI), and 8.15 ± 9.37 (TII), with no significant differences between groups (*p* = 0.63). Physical pain, psychological discomfort and disability presented highest OHIP-14-dimension score, while functional limitation and social disability exhibited the lowest score indicating that self-consciousness, tension and embarrassment as well as painful aching and limitations in eating were the factors which were significantly affected within the groups (Fig. 2A–C).

**Table 4:** Percentage distribution of responses to each question of OHIP-14 questionnaire

Question	Autogenous bone graft						1:1 autogenous bone graft and DPBM						1:1 autogenous bone graft and BBGM					
	0	1	2	3	4	Mean	0	1	2	3	4	Mean	0	1	2	3	4	Mean
Q1	90%	10%				0.10	90%	10%				0.10	80%	5%	15%			0.35
Q2	90%		10%			0.20	90%		5%		5%	0.30	85%	5%	5%	5%		0.30
Q3	65%	10%	25%			0.60	20%	40%	35%	5%		1.25	40%	45%	10%		5%	0.85
Q4	35%	15%	25%	25%		1.40	40%	10%	25%	10%	15%	1.50	45%	25%	30%			0.85
Q5	50%	10%	10%	5%	25%	1.45	60%	10%	10%	15%	5%	0.95	50%	15%	15%	10%	10%	1.15
Q6	55%	15%	10%	15%	5%	1.00	45%	15%	25%	15%		1.10	60%	20%	10%		10%	0.80
Q7	70%	25%			5%	0.45	55%	15%	25%		5%	0.85	70%	20%	5%		5%	0.50
Q8	70%	20%	5%		5%	0.50	70%	20%	5%	5%		0.45	75%	20%	5%			0.30
Q9	60%	15%	20%	5%		0.70	60%	15%	20%	5%		0.70	75%	10%	5%	5%	5%	0.55
Q10	45%	15%	10%	15%	15%	1.40	65%	10%	5%	15%	5%	0.85	55%	20%	15%		10%	0.90
Q11	70%	20%	10%			0.40	60%	20%	20%			0.60	85%		15%			0.30
Q12	90%	10%				0.10	95%	5%				0.05	85%	5%	10%			0.25
Q13	60%	10%	25%	5%		0.75	55%	15%	10%	20%		1.15	70%	5%	15%		10%	0.75
Q14	80%	15%	5%			0.25	90%	10%				0.10	80%	10%	10%			0.30
	Total OHIP-14 score: 186						Total OHIP-14 score: 199						Total OHIP-14 score: 163					
	Mean OHIP-14 score for each patient: 9.30 (SD 9.25)						Mean OHIP-14 score for each patient: 9.95 (SD 7.96)						Mean OHIP-14 score for each patient: 8.15 (SD 9.37)					
	Mean OHIP-14 score for all items: 0.66 (SD 0.48)						Mean OHIP-14 score for all items: 0.71 (SD 0.48)						Mean OHIP-14 score for all items: 0.58 (SD 0.30)					

0 = never; 1 = hardly ever or nearly never; 2 = occasionally; 3 = fairly often or many times; 4 = very often

*BBGM* biphasic bone graft material, *DPBM* deproteinized porcine bone mineral, *SD* standard deviation

Questionnaires after 1 week revealed moderate influence on patient’s daily life activities with all treatment modalities (Tables 5, 6, 7). Most patients were satisfied with the treatment and would recommend it to friends and relatives (Table 5). The average numbers of days on sick leave or been off work were 0.5 (SD 1.2), and physical activity were affected for 1.7 days (SD 3.3), with no significant differences between groups. The average number of days with diminished eating and speaking ability as well as sleep impairment were 2.7 (SD 2.5), 0.4 (SD 1.4), and 0.6 (SD 1.3), with no significant differences between groups (Table 7).

**Table 5:** Questionnaire assessing social and working isolation, physical appearance and quality of life alterations, at 1 week

Question	Autogenous bone graft		1:1 autogenous bone graft and DPBM		1:1 autogenous bone graft and BBGM	
	Yes %	No %	Yes %	No %	Yes %	No %
Social isolation						
Did you keep your usual social activities?	95	5	85	15	80	20
Have you continued practicing your favorite sport or hobbies?	90	10	75	25	60	40
Working isolation						
Did you ask for sick leave or discontinue your work?	10	90	20	80	35	65
Did the surgery affect your performance at work?	10	90	10	90	15	85
Did anyone accompany you or drive you to work due to surgery?		100		100	10	90
Has this person discontinued his/her work to do so?		100		100	5	95
Did somebody accompany you for suture removal?	10	90	5	95	10	90
Physical appearance						
Have you noticed changes in your physical appearance?	25	75	40	60	30	70
Is it what you expected?	100		100		85	15
Has it been worse than expected?	10	90	5	95	15	85
Has it been better than expected?	65	35	90	10	60	40
Mean duration of the quality of life alterations						
Are you satisfied with the treatment?	100		100		100	
Would you recommend it?	100		100		95	5
Would you repeat it?	95	5	95	5	95	5

*BBGM* biphasic bone graft material, *DPBM* deproteinized porcine bone mineral

**Table 6:** Questionnaire assessing eating and speaking ability, diet variations, sleep impairment, pain and discomfort, at 1 week

Question	Autogenous bone graft				1:1 autogenous bone graft and DPBM				1:1 autogenous bone graft and BBGM			
	0 %	1 %	2 %	3 %	0 %	1 %	2 %	3 %	0 %	1 %	2 %	3 %
Eating ability and diet variations												
Did you continue with your usual diet?	20	30	45	5	15	30	45	10	25	35	40	
Did you notice any change in the perception of taste?	70	20	10		65	20		15	75	10	10	5
Did you notice any change in chewing ability?	25	10	40	25	25	15	25	35	25	20	25	30
Did you have problems opening your mouth?	50	10	10	30	55	5	15	25	45	15	15	25
Speaking ability noticed												
Have you notice any change in voice?	75	20		5	70	25	5		80	5	10	5
Have you notice any change in your ability to speak?		95	5		70	25	5		75	10	5	10
When you talk with other people, do they understand you?	70	25	5		15	85			5	80	10	5
Sleep impairment												
Have you had problems falling sleep?	65	20	5	10	80	10	5	5	70	25		5
Have you experienced interruptions in sleep?	5	80	15		75	20		5	60	20	5	15
Have you felt drowsy?	55	30	5	10	50	30	15	5	55	30	10	5
Pain and discomfort at suture removal												
Has the removal of suture been uncomfortable?	75	10		15	80	5	10	5	90	10		
Has the appointment for suture removal caused you anxiety?	95			5	95		5		95	5		

0 = not at all; 1 = close to normal; 2 = almost normal; 3 = a little

*BBGM* biphasic bone graft material, *DPBM* deproteinized porcine bone mineral

**Table 7:** Questionnaire assessing days of recovery, at 1 week

Question	Autogenous bone graft	1:1 autogenous bone graft and DPBM	1:1 autogenous bone graft and BBGM	*p*-value
	Mean (range), SD	Mean (range), SD	Mean (range), SD	
How many days have you been on sick leave or been off work?	0.2 (0–3), 0.7	0.5 (0–7), 1.6	0.9 (0–3), 1.2	0.20
How many days have you had eating difficulties?	2.4 (0–7), 2.3	3.1 (0–11), 3.0	2.6 (0–7), 2.3	0.67
How many days have you had speech difficulties?	0.4 (0–7), 1.6	0.5 (0–7), 1.6	0.4 (0–3), 0.8	0.97
How many days has your sleep been affected?	1.1 (0–6), 1.8	0.4 (0–3), 0.8	0.4 (0–3), 0.8	0.12
How many days has your physical activity been affected?	0.9 (0–4), 1.4	1.9 (0–7), 2.5	2.3 (0–21), 4.9	0.39

*BBGM* biphasic bone graft material, *DPBM* deproteinized porcine bone mineral, *MSFA* maxillary sinus floor augmentation,* SD* standard deviation

*Statistically significant

Questionnaires after 1 month disclosed fast recovery with all treatment modalities (Table 8). The average number of days with pain were 3.5 (SD 3.9), with no significant differences between groups. VAS score of surgical impact on performing daily work, eating and speaking ability as well as sleep impairment were 17.3 (SD 32.2), 55.0 (42.5), 6.3 (SD 20.5), and 19.1 (34.3), with no significant differences between groups (Table 8).

**Table 8:** Questionnaire assessing pain, sick leave, performance, ability to eat, sleep and speak, at 1 month

Question	Autogenous bone graft	1:1 autogenous bone graft and DPBM	1:1 autogenous bone graft and BBGM	*p*-value
	Mean (range), SD	Mean (range), SD	Mean (range), SD	
In how many days have you had pain after surgery?	3.1 (0–8), 2.4	3.1 (0–10), 2.9	4.2 (0–20), 5.7	0.60
In how many days have you been on sick leave from daily activities such as work, school, etc., due to pain?	0.2 (0–3), 0.7	1.0 (0–7), 2.0	1.0 (0–4), 1.5	0.16
Did the operation affect your performance of your daily work? (VAS: 0–100)	20.2 (0–100), 35.1	13.8 (0–100), 28.3	18.0 (0–100), 34.1	0.82
In how many days have you been affected in your work?	1.4 (0–7), 2.2	1.5 (0–7), 2.4	0.9 (0–4), 1.4	0.61
Have you been able to eat a normal diet in the postoperative period? (VAS: 0–100)	58.4 (0–100), 41.4	50.5 (0–100), 45.2	56.1 (0–100), 42.6	0.84
In how many days have you been unable to eat your normal diet?	3.3 (0–14), 3.4	3.7 (0–14), 3.7	2.2 (0–7), 2.2	0.31
Have you noticed changes in your speech after surgery? (VAS: 0–100)	3.3 (0–58), 12.9	5.3 (0–73), 17.4	10.2 (0–100), 28.5	0.56
In how many days have you noticed changes in your speech?	0.4 (0–7), 1.6	1.5 (0–14), 4.3	0.4 (0–3), 0.8	0.33
Have you had trouble sleeping at night after surgery? (VAS: 0–100)	15.0 (0–100), 32.6	21.2 (0–100), 36.2	21.0 (0–100), 35.4	0.81
In how many days have your night's sleep been affected?	0.9 (0–7), 1.8	1.2 (0–7), 2.3	0.7 (0–5), 1.3	0.69

*BBGM* biphasic bone graft material, *DPBM* deproteinized porcine bone mineral, *MSFA* maxillary sinus floor augmentation, *SD* standard deviation, *VAS* visual analogue scale (0 = minimal to 100 = maximum)

**Fig. 3:**
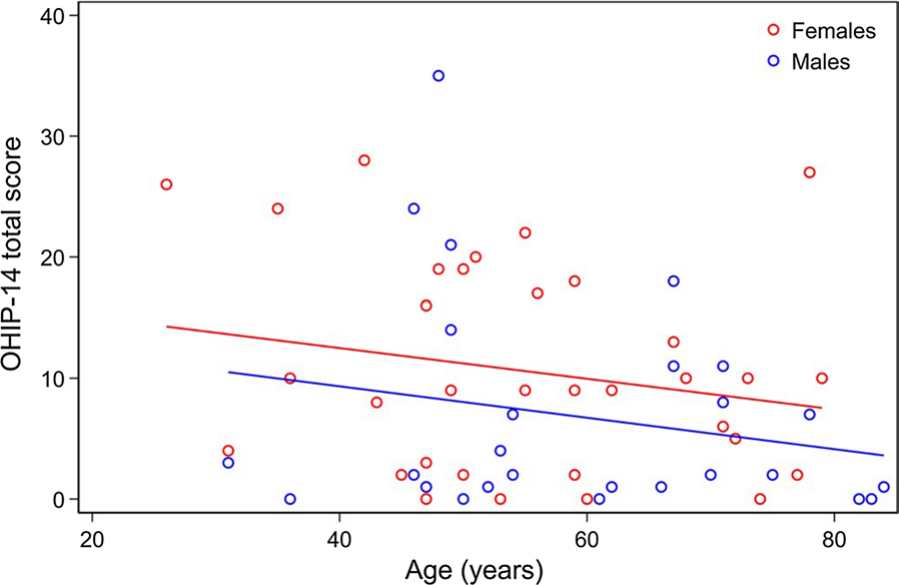
OHIP-14 scores at enrollment correlated to age and gender

Correlation between impaired OHRQoL (OHIP-14 score ≥ 10) at enrollment and patient´s perception of recovery revealed that number of days on sick leave or been off work were significantly increased with impaired OHRQoL (0.4 days, SD 0.3) compared with unimpaired OHRQoL (0.0 days, SD 0.0) (*p* < 0.001) in the CG, whereas no significant difference was found in TI or TII (Table 9). In general, patient’s perception of recovery seems to be unaffected by OHRQoL at enrollment, although patients with impaired OHRQoL reported significantly more working days affected by the surgical intervention in TI (*p* = 0.04) and higher VAS scores of sleep disturbance in CG (*p* = 0.02). Nevertheless, patients with impaired OHRQoL generally revealed a higher but not significant score in most of the parameters surveyed (Table 9). Females and younger age generally reported higher OHIP-14 score at enrollment, although no significant correlation between a higher OHIP-14 score at enrollment and age (*p* = 0.13) or gender (*p* = 0.18) was observed (Fig. 3). Moreover, no significant correlation between higher OHIP-14 score at enrollment and numbers of days with pain (*p* = 0.48) or on sick leave (*p* = 0.28) was observed.

**Table 9:** Correlation between presurgical OHIP-14 item score and patient’s perception of recovery

Question	Autogenous bone graft			1:1 autogenous bone graft and DPBM			1:1 autogenous bone graft and BBGM		
	OHIP-14 score < 10 (no.: 12) Mean, range, SD	OHIP-14 score ≥ 10 (no.: 8) Mean, range, SD	*p*-value	OHIP-14 score < 10 (no.: 10) Mean, range, SD	OHIP-14 score ≥ 10 (no.: 10) Mean, range, SD	*p*-value	OHIP-14 score < 10 (no.: 15) Mean, range, SD	OHIP-14 score ≥ 10 (no.: 5) Mean, range, SD	*p*-value
Week									
Days, sick leave or off work?	0.0 (0–0), 0.0	0.4 (0–3), 0.3	0.00*	0.2 (0–2), 0.6	0.8 (0–7), 2.2	0.42	0.9 (0–3), 1.3	0.8 (0–2), 1.1	0.88
Days, eating difficulties?	2.3 (0–6), 2.5	2.5 (0–7), 2.6	0.86	3.1 (0–11), 3.5	3.0 (0–7), 2.6	0.94	2.5 (0–7), 2.3	2.8 (0–6), 2.4	0.81
Days, speech difficulties?	0.1 (0–1), 0.3	0.9 (0–7), 2.5	0.28	0.7 (0–7), 2.2	0.2 (0–2), 0.6	0.50	0.3 (0–3), 0.8	0.4 (0–2), 0.9	0.82
Days, sleep affected?	1.0 (0–3), 1.7	1.1 (0–6), 2.2	0.91	0.0 (0–0), 0.0	0.7 (0–3), 1.1	0.06	0.3 (0–3), 0.8	0.4 (0–2), 0.9	0.82
Days, physical activity affected?	0.6 (0–3), 1.1	1.3 (0–4), 1.8	0.29	1.5 (0–7), 2.6	2.3 (0–7), 2.5	0.49	2.8 (0–21), 5.5	1.3 (0–3), 1.3	0.56
Month									
Days, pain after surgery?	2.3 (0–8), 2.4	4.1 (1–7), 2.2	0.11	2.0 (0–7), 2.2	4.1 (1–10), 3.3	0.11	4.3 (0–20), 6.4	3.8 (0–7), 2.8	0.87
Days, sick leave or off work?	0.0 (0–0), 0.0	0.4 (0–3), 1.1	0.22	0.3 (0–2), 0.7	1.6 (0–7), 2.6	0.14	1.1 (0–4), 1.6	0.8 (0–2). 1.1	0.70
Work affected, (VAS)	17.6 (0–100), 34.5	24.1 (0–100), 37.9	0.70	12.9 (0–100), 31.1	14.7 (0–79), 26.9	0.89	17.1 (0–100), 34.1	20.4 (0–86), 37.3	0.86
Days, work affected?	1.5 (0–7), 2.5	1.1 (0–3), 1.6	0.69	0.4 (0–3), 1.0	2.6 (0–7), 3.0	0.04*	0.7 (0–3), 1.2	1.4 (0–4), 1.9	0.34
Eating difficulties, (VAS)	62.6 (0–100), 42.6	52.1 (0–98), 41.5	0.59	52.7 (0–100), 48.3	48.3 (0–100), 44.5	0.83	56.8 (0–100), 42.6	54.0 (0–100), 47.5	0.90
Days, eating difficulties?	3.3 (0–14), 4.0	3.1 (0–7), 2.2	0.90	2.6 (0–10), 3.4	4.7 (1–14), 3.9	0.22	1.9 (0–7), 2.0	2.8 (0–7), 2.8	0.44
Speech changes, (VAS)	0.1 (0–1), 0.3	8.1 (0–58), 20.2	0.18	3.3 (0–31), 9.8	7.3 (0–73), 23.1	0.62	7.8 (0–100), 25.7	17.2 (0–86), 38.5	0.54
Days, speech changes?	0.0 (0–0), 0.0	0.9 (0–7), 2.5	0.22	1.4 (0–14), 4.4	1.5 (0–14), 4.4	0.96	0.4 (0–3), 0.9	0.2 (0–1), 0.4	0.64
Sleep disturbances? (VAS)	2.2 (0–14), 4.7	34.3 (0–100), 44.5	0.02*	13.0 (0–100), 32.0	29.3 (0–100), 40.0	0.33	14.9 (0–97), 27.5	39.4 (0–100), 52.2	0.19
Days, sleep affected?	0.4 (0–3), 1.0	1.7 (0–7), 2.6	0.13	0.8 (0–7), 2.2	1.6 (0–7), 2.4	0.45	0.7 (0–5), 1.4	0.6 (0–2), 0.9	0.88

*BBGM* biphasic bone graft material, *DPBM* deproteinized porcine bone mineral, *OHIP-14* Oral Health Impact Profile-14, *SD* standard deviation, *VAS* visual analogue scale (0 = minimal to 100 = maximum)

*Statistically significant

## Discussion

Patient’s perception of recovery after MSFA with autogenous bone graft harvested from the zygomatic buttress was compared with 1:1 mixture of autogenous bone graft and DPBM or BBGM using validated self-administrated questionnaires including a correlation with presurgical OHRQoL. A total of 60 partially edentulous healthy patients with a missing posterior maxillary tooth were included and randomly allocated into three groups of 20 patients. All included patients attended postsurgical examinations and completed questionnaires and VAS. High treatment satisfaction and willingness to undergo the same type of surgery or recommend the treatment to friends and relatives were reported by 93%. The average number of days with pain or on sick leave were 3.5 (SD 3.9) and 0.5 (SD 1.2), with no significant differences between groups. Inability to eat seems to be the largest obstacle within the groups. However, no significant differences were observed in terms of surgical impact on performing daily work, eating and speaking ability as well as sleep impairment. Patient’s perception of recovery seems to be unaffected by OHRQoL at enrollment. Nevertheless, patients with impaired OHRQoL generally reported higher but not significant score in most of the parameters surveyed. Consequently, MSFA with diminutive autogenous bone graft harvesting is associated with high patient satisfaction, limited postoperative discomfort and willingness to undergo similar surgery. Presurgical OHRQoL, gender or age seems not to be significantly associated with impaired patient´s perception of recovery.

MSFA with autogenous bone graft used alone or in combination with different ratios of bone substitutes generates more newly formed bone, higher bone-to-implant contact, and earlier bone formation compared with the use of a bone substitute alone [13, 29–31]. However, harvesting of extraoral and intraoral autogenous bone graft negatively influences postsurgical OHRQoL due to increased pain, impaired eating and speaking ability, sleep impairment, limitation in daily routine, and sick leave [12, 32–34]. Patient’s perception of recovery is therefore strongly associated with the surgical procedure, absence of pain, rapidly recovery of oral function and return to normal lifestyle. From a patient perspective, it would be an advantage, if postoperative discomfort could be lessened by diminishing the need for extensive autogenous bone graft harvesting. Previous studies have revealed successfully implant treatment outcome following MSFA with autogenous bone graft harvested from the surgical site involving the zygomatic buttress or buccal sinus wall [35, 36]. Moreover, mild to moderate pain and inability to participate in daily activities for 2 and 3 days have been reported after MSFA with a 1:1 mixture of autogenous bone graft harvested from the buccal antrostomy and a bone substitute as evaluated by questionnaire, five-point Likert-type scale and VAS [15]. Moreover, low postsurgical discomfort and moderate values of pain during the first two days (< 50) as estimated by VAS, with a tendency to progressively decrease over the next two days have been reported following MSFA with a bone substitute alone [21]. These results seem to be in accordance with the present study indicating that MSFA with diminutive autogenous bone graft from the surgical site or use of a bone substitute alone causes pain for approximately 3–4 days.

Postoperative pain is a common cause for sick leave or been off work. Previous studies have reported that pain was most pronounced on the first postoperative day and significantly declined to presurgical values after 3–7 days following MSFA with a bone substitute alone as evaluated by questionnaire and VAS [18–20]. Moreover, the median sick leave was 5 days, and more than half of the patients had 3 days off work and 10–20% had not returned to work after 1 week [19]. In the present study, the average number of days with pain following MSFA was 3.5 and number of days on sick leave or been off work was less than one day. Consequently, the use of diminutive autogenous bone graft from the zygomatic buttress seems not to deteriorate the number of days with pain or on sick leave following MSFA as compared with the use of a bone substitute alone.

Physical limitations on daily activities significantly influence patient´s perception of recovery. Previous studies have described inability to participate in routine daily activities and interference with general activities for approximately 3–5 days following MSFA with a bone substitute alone [18, 19]. In the present study, limitations of physical activity as well as sleep impairment, eating and speech difficulties varied between 1 and 4 days, with no significant differences between groups. Consequently, harvesting of diminutive autogenous bone graft from the zygomatic buttress in conjunction with MSFA seems not to cause further limitations in daily activities as compared with the use of a bone substitute alone.

Patient´s willingness to undergo same type of surgery is essential for future decision-making process. Previous studies have reported that most of the patients indicated willingness to repeat the surgical intervention if needed and would recommend the treatment to friends and relatives following MSFA with autogenous bone graft or a bone substitute alone [16, 18, 20]. These results are in accordance with the present study. Moreover, more than half of the patients in the present study indicated that the surgical intervention was better than expected. Consequently, MSFA in conjunction with diminutive harvesting of autogenous bone graft is associated with high treatment satisfaction, surgical intervention better than expected, and willingness to undergo same type of surgery, if needed.

Patient’s concerns of pain, complications, donor site morbidity and influences on normal lifestyle are important criteria for selection of a specific donor site in elective preprosthetic surgery. The incidence and severity of complications in conjunction with MSFA are generally low [1, 5], whereas harvesting of extensive extraoral and intraoral autogenous bone graft is associated with risk of irreversible disabling complications including endodontic therapy of teeth adjacent to the donor site, neurosensory disturbances of the skin as well as numbness or altered sensation of the lower lip, chin and oral mucosa [11, 37]. Donor site morbidity as well as intraoperative and postoperative complications in conjunction with harvesting of autogenous bone graft from the zygomatic buttress or buccal sinus wall seems to be negligible [35, 36], which is in accordance with the present study. However, the amount of autogenous bone graft that can be harvested from the zygomatic buttress is limited. Though, a previous study has showed that harvesting of autogenous bone graft from the zygomatic buttress enables successful placement of 1–3 implants in conjunction with MSFA [35].

Patient´s perception of recovery is associated with the expectation and experience of the surgical intervention and related complications. Smoking, increasing age, poor oral hygiene, and history of periodontitis are well-known risk factors for intraoperative and postoperative complications in conjunction with MSFA and harvesting of intraoral autogenous bone graft [37–39]. The frequency and severity of complications in the present study were negligible. However, correlation between smoking habits, age, oral hygiene or reason for tooth loss and complications were not conducted.

Patient’s perception of recovery is also influenced by patient-related predictors, past dental experiences as well as psychological and psychosocial factors, which are rarely assessed [22–26]. Psychologic distressed, high level of anxiety and impaired perception of OHRQoL seem to have a negative impact on postsurgical recovery [22–26]. In the present study, no unambiguous association between impaired presurgical OHRQoL and patient’s perception of recovery was revealed. However, patients with impaired presurgical OHRQoL generally reported higher but not significant score in most of the parameters surveyed. Furthermore, females and younger age reported higher OHIP-14 score at enrollment, which is in accordance with a previous study concluding that females and younger age are patient-related predictors for delayed recovery following MSFA [19]. A significant correlation between impaired OHRQoL at enrollment and numbers of days on sick leave following MSFA with autogenous bone graft were identified. However, the difference seems to have little clinical relevance.

The present study is characterized by various limitations including small patient sample, solely collecting postsurgical information corresponding to 1 week and 1 month, and no systematic registration of quantity and period of need for analgesics. Moreover, correlation between patient’s perception of recovery and socioeconomic status, educational background, and level of daily physical functioning were not performed. Conclusions drawn from the results of this study should therefore be interpreted with caution and the above-mentioned aspects are recommended to be incorporated in future studies assessing patient´s perceptions of recovery following MSFA.

## Conclusion

Within the limitations of the present study, it can be concluded that MSFA with autogenous bone graft from the zygomatic buttress used alone or in a 1:1 mixture with DPBM or BBGM is associated with high treatment satisfaction and willingness to undergo the same type of surgery or recommend the treatment to friends and relatives. MSFA including harvesting of autogenous bone graft from the surgical site is associated with 3–4 days of pain and less than one day on sick leave. No significant difference was observed between groups in terms of eating and speaking ability, physical appearance and sleep impairment. Impaired presurgical OHRQoL, age or gender seems not predispose for delayed recovery, although patients with impaired OHRQoL generally reported a higher score in most of the parameters surveyed.

## Data Availability

Study protocol and all data are available from the corresponding author on reasonable request.

## Abbreviations

BBGM: Biphasic bone graft material
CG: Control group
DPBM: Deproteinized porcine bone mineral
MSFA: Maxillary sinus floor augmentation
OHIP: Oral Health Impact Profile
OHRQL: Oral Health-related Quality of Life
SD: Standard deviation
TI: Test group I
TII: Test group II
VAS: Visual analogue scale
